# Book Reviews

**Published:** 1896-04

**Authors:** 


### BOOK REVIEWS.

The Functional Examination of the Eye. By John Herbert Claiborne, Jr., M.D., New York. The Edwards & Docker Company, Philadelphia.

This little book is just before us, and for fear that others like ourselves may not understand just why its author produced it, we will quote the closing lines of the introduction: "The book is a result of a course of lectures delivered for a number of years at the New York Polyclinic, and more particularly of a course in practical instruction given by the author in the College of Physicians and Surgeons, New York."

With such clear, practical, and concise little books on Refraction as those by Valk, Hartridge, Tiffany, and others, it is difficult to see how any one could fall into the error of publishing even a little book upon this subject, unless its contents were to be marked by some distinct originality.

Had the author of the present little book confined the copies of the same to the pupils who had listened to his instruction, then there might be some reason for the publication of these lectures, but how the medical profession could be benefited, and especially ophthalmologists, by perusing their pages, we fail to comprehend.

The lectures are gotten up in a very neat and handy form, and the print is unusually clear and good. A few typographical errors somewhat mar its excellency. For instance, on page 55 we read "cylindrical glasses *reflect* only those rays," etc. We readily understand that the author does not hold that the rules thus laid down by him are sufficient for fitting lenses to eyes, but that they apply only to the test cards and trial lenses as one step in the thorough examination. Of course to those who are familiar with refractive work the present book cannot possibly do any harm, for they will absorb only the good portions of it; but to those who are not so fortunate, we think that some of the teachings might lead such astray. We refer, for instance, to the author's method of fitting lenses for myopic astigmatism, as described on page 60.

The fallacy in this method lies in the fact that hyperopic frequently simulates myopic astigmatism, which can only be revealed by retinoscopy, the ophthalmoscope, or some mydriatic. We hold that no eyes, with the exception of presbyopic, should be fitted by test lenses *alone*.

It was not our intention to make such a marked criticism on our review, but honesty demands that reviewers should try and be truthful instead of lauding every book to the profession, w..ether it be good, bad, or indifferent. As written, the author has certainly placed it in attractive form, and we trust his labors will not be entirely unrewarded. D. R.

---

SYPHILIS IN THE MIDDLE AGES AND IN MODERN TIMES. By Dr. F. Buret, Paris, France. Translated from the French, with notes, by A. H. Ohmann-Dumesnil, M.D., Professor of Dermatology and Syphilology in the Marion Sims College of Medicine. Being Volumes II. and III. of "Syphilis To-day and among the Ancients," complete in three volumes. Extra Cloth, $1.50 net. The F. A. Davis Co., Publishers, 1914 and 1916 Cherry street, Philadelphia.

The completion of this interesting work, the first volume of which attracted such favorable attention three or four years ago, will be very welcome. By these historical researches Dr. Buret has certainly thrown a flood of light upon the ancient history of syphilis, and has well succeeded in clearing up a great deal that was uncertain, traditional, or even mythical.

The first volume discussed syphilis in ancient and prehistoric times. Here the author demonstrated the evidences of syphilis in prehistoric remains, and traced the disease to the most ancient historic epochs. He says that syphilis never sprang into being *de novo,* and never died out. Coming to the middle ages and modern times (in the present volume) the author includes the history of the disease up to the siege of Naples by the French king, Charles VIII. (1495), and from that time to the present. During the middle ages, through a period of about one thousand years, syphilis always existed in an endemic state, especially in places of debauchery.

From time to time it disseminated itself in different countries through large movements of people. Current leprosy, which once included a variety of skin affections, finally came to designate a venereal disease acquired by coitus. The common leprosy of that time was syphilis. Therefore the disease could not have burst upon the world anew at the siege of Naples in either of the opposing armies, although the wars of that time, with attending immorality and disregard of hygiene, furnished the most favorable opportunities for its dissemination. Of course America is vindicated of the charge, once taught, of having infected the old world through the persons of Columbus and his followers.

The author has ransacked history and classical literature for material for this book, and his results will be a monument to him. Any one can read this work with profit; no one at all interested in the subject of syphilis should fail to read it. The author has made the entire medical profession his debtor. The translation is excellent, preserving almost entirely the pleasing style of the author. While some of the author's conclusions may appear ultra, or otherwise open to criticism, we think he has established beyond question his leading proposition, that *syphilis is a contemporary of all ages.*

---

AN ATLAS OF THE NORMAL AND PATHOLOGICAL NERVOUS SYSTEMS, together with a Sketch of the Anatomy, Pathology and Therapy of the Same. By Dr. Christfried Jakob, practicing physician in Bombay, formerly first assistant in the Medical Clinic at England. Translated by Jos. Collins, M.D., Instructor of Nervous Diseases, New York Post-Graduate Medical College, etc., etc. Published by William Wood & Co., New York.

The author has divided the atlas into six sections. Section I. treats of the morphology of the nervous system. Sec. II. elaborates the development and structure of the nervous system. Sec. III., on the topographical anatomy of the brain, cord, and peripheral nerves in serial plates. Secs. IV. and V. treat of the general and special pathology of the cerebro-spinal system.

These five sections are all demonstrated by means of plates, which are most exquisite, both from anatomical and artistic standpoint. The text explanatory of the plates is lucid and very comprehensible. That the intricate anatomy of the brain and cord have been so distinctly and graphically demonstrated is indeed an achievement in histological anatomy.

Up to the present, this work is the most complete that the writer has seen, and is, in his opinion, destined to be an authority on the cerebral and spinal anatomy.

In Section VI. some valuable points on the technique of an autopsy are given, the study and observation of which will be of much service to all workers in this field.

Had the author stopped here, I think the work would have been complete, without the appendix of the laconic and very limited synopsis of the treatment of diseases of the nervous system. The subject-matter of this chapter is too great and important to be discussed with a few desultory remarks, and we think it were better had the treatment been left out; not that what is given is not worthy, but too incomplete. The translator has doubtless done the original justice, which is to say that he too has wrought with skilled hands and clear intelligence. The publishers are to be praised for the neatness and agreeable execution of this work. H. H.

---

MEDICAL JURISPRUDENCE, FORENSIC MEDICINE, AND TOXICOLOGY. By R. A. Witthaus, A.M., M.D., and Tracy C. Becker, A.B., LL.B., and a staff of collaborators. In four royal octavo volumes. Volume III., Forensic Medicine (continued). William Wood & Company, New York.

The third volume of this excellent work continues the general subject of Forensic Medicine commenced in Volume II. The first article is by Dr. J. H. Woodward, on "Vision and Audition in their Medico-Legal Relations." The subject is an extremely important one, particularly to the physician in his capacity of expert, since derangements of sight or hearing, real or feigned, play a most important part in many lawsuits.

The next paper is on "The Medico-Legal Aspect of Insurance," and this topic has been ably handled by David Murray, Esq., and G. J. Edwords, Esq., well-known experts in this line.

The next section on "Insanity in its Relations to Medical Jurisprudence," by Dr. E. D. Fisher, is the largest article in the book, occupying over 200 pages, and its author's well-known reputation in this branch of medicine is sufficient guaranty of the value of his contribution.

The next paper, on "Mental Unsoundness in its Legal Relations," is by T. C. Becker, Esq., one of the associate editors of this work, and, coming from the pen of a practical lawyer, is a most valuable contribution to the literature of the subject.

The last section is on "The Care and Custody of Incompetent Persons," by the Honorable Goodwin Brown. The article is a perfect epitome of the legal and medical sides of this subject, with an abstract of the laws of the various States that bear upon it.

The third volume amply justifies the promise that this set of books will, when completed, be of exceptional, if not of indispensable, value both to the practical physician and to the lawyer.

---

PRENANCY, LABOR AND THE PUERPERAL STATE. By Egbert H. Grandin, M.D., and George W. Jarman, M.D., Obstetric Surgeons to the New York Maternity Hospital, New York City. Illustrated with forty-one photographic plates. Published by The F. A. Davis Company, 1916 Cherry St., Philadelphia.

To-day, the authors state, the major part of obstetric practice is founded on *fact*. The general practitioner, amidst the activities of his calling, when in search of information, wishes to secure it without the loss of time entailed in searching through a mass of theory and statistics. On such grounds the present work has been prepared. It aims at being a guide to practice. It is clinical in its teaching. Anatomical and embryological and pathological data are inserted only when essential.

It appears upon close examination of the text that these aims of the authors have been very well carried out. The work is essentially practical, containing a concise description of normal pregnancy and normal labor untrammelled of theoretical or statistical discussions. Herein it will prove of special usefulness to the student and young practitioner. The half-tone photographic plates are original and very good, though some of them, it seems to us, are unnecessary. With the volume on "Obstetric Surgery," by the same authors, this book will constitute their *Practical Obstetrics*, which is thoroughly deserving of a high place in our obstetrical literature.

A HANDBOOK OF MEDICAL DIAGNOSIS. By James B. Herrick, M.D., Adjunct Professor of Medicine, Rush Medical College, Chicago. In one handsome 12mo volume of 429 pages, with 80 engravings and 2 colored plates. Cloth, $2.50. Lea Brothers & Co., Publishers, Philadelphia.

While it is stated that this handbook is intended especially for students, we think it will be found equally useful for practitioners. Very properly the author emphasizes the importance of a diagnosis as a prerequisite condition to intelligent treatment of a given case. The ability to make an accurate diagnosis is a matter of training and experience. The book of Dr. Herrick presents in a clear and concise manner the most important and conclusive features of diseases which must be considered in reaching a diagnosis. The work is divided, like usual works on Practice, into Infectious Diseases, Diseases of the Respiratory System, of the Circulatory System, of the Nervous System, Constitutional Diseases, etc., and all these diseases are discussed fully enough for all practical purposes.

Practitioners will find this a convenient and reliable working manual of medical diagnosis.

---

HOW TO EXAMINE FOR LIFE INSURANCE. By John M. Keating, M.D., late Medical Director of Penn Mutual Life Insurance Company; Ex-President of the Association of Life Insurance Medical Directors, etc. Third edition. Published by W. B. Saunders, 925 Walnut St., Philadelphia. Price $2.00.

Those who do much work in life insurance will often feel the need of such a book as this. Indeed, it is almost a necessity. The work of Dr. Keating has long enjoyed a popularity in this field, and for the medical examiner's purposes it certainly leaves little, if anything, to be desired. The first part of the book has to do especially with the applicant, discussing the methods of examination of lungs, heart, kidneys, etc., and the results of said examination in their relation to the quality of the "risk." Examiners will find in this part much to interest and instruct. The second part consists of "Instructions to Medical Examiners as Issued by Various Life Insurance Companies," which gives under each company the rulings of the home office on "risks" impaired from one cause or another. It gives us pleasure to recommend this book to every medical examiner.

MANUAL OF MEDICAL JURISPRUDENCE AND TOXICOLOGY. By Henry C. Chapman, M.D., Professor of Medical Jurisprudence in Jefferson Medical College, Philadelphia, etc. Second edition, revised. Published by W. B. Saunders, 925 Walnut St., Philadelphia. Price $1.50.

The author's experience as teacher of medical jurisprudence and as coronor's physician for the city of Philadelphia for a number of years enables him to place before the general practitioner a manual of medical jurisprudence and toxicology containing all the essential principles of these subjects without narrating illustrative cases. The manual is concise and well written, embracing all the subjects usually treated under these heads. The book contains fifty-five illustrations and three colored plates.

---

DIET IN SICKNESS AND IN HEALTH. By Mrs. Ernest Hart, formerly student of the Faculty of Medicine of Paris, and of the London School of Medicine for Women. With an introduction by Sir Henry Thompson, London. Published by W. B. Saunders, Philadelphia.

This book, comprising 215 pages, sets forth accurately and concisely the chemistry and physiology of dietetics.

Treats of the physiology and pathology of digestion, with illustrations. Gives also physiological indications for special dietary.

It is well termed by Sir Henry Thompson, "A handbook on food and feeding in health and disease," and supplies an important place in the literature of dietetics. W. J. B.

---

MISKEL. A novel. By L. M. Phillips, M.D., New York. Price, 50 cents. Published by Bailey & Fairchild Company, New York.

This is the second in the series of "Doctors' Stories" to be issued quarterly by this company. It is supposed to be of special interest to physicians from the important part which hypnotism, mental telepathy, etc., play in a very impossible plot. The author seems to possess some of the weird imagination of Rider Haggard without the latter's ability to write a story. Physicians will not find this medicated novel either interesting or edifying.

